# Quantitative measures and 3D shell models reveal interactions between bands and their position on growing snail shells

**DOI:** 10.1002/ece3.7517

**Published:** 2021-05-02

**Authors:** Hannah J. Jackson, Jenny Larsson, Angus Davison

**Affiliations:** ^1^ School of Life Sciences University of Nottingham Nottingham UK; ^2^ Department of Animal and Plant Sciences University of Sheffield Sheffield UK

**Keywords:** banding, biomineralization, *Cepaea*, mollusc, Turing

## Abstract

The nature of shell growth in gastropods is useful because it preserves the ontogeny of shape, colour, and banding patterns, making them an ideal system for understanding how inherited variation develops, is established and maintained within a population. However, qualitative scoring of inherited shell characters means there is a lack of knowledge regarding the mechanisms that control fine variation. Here, we combine empirical measures of quantitative variation and 3D modeling of shells to understand how bands are placed and interact. By comparing five‐banded *Cepaea* individuals to shells lacking individual bands, we show that individual band absence has minor but significant impacts upon the position of remaining bands, implying that the locus controlling band presence/absence mainly acts after position is established. Then, we show that the shell grows at a similar rate, except for the region below the lowermost band. This demonstrates that wider bands of *Cepaea* are not an artifact of greater shell growth on the lower shell; they begin wider and grow at the same rate as other bands. Finally, we show that 3D models of shell shape and banding pattern, inferred from 2D photos using ShellShaper software, are congruent with empirical measures. This work therefore establishes a method that may be used for comparative studies of quantitative banding variation in snail shells, extraction of growth parameters, and morphometrics. In the future, studies that link the banding phenotype to the network of shell matrix proteins involved in biomineralization and patterning may ultimately aid in understanding the diversity of shell forms found in molluscs.

## Graphical abstract

The qualitative scoring of snail shell banding means there is a lack of knowledge regarding the mechanisms that control fine variation. Here, we combine empirical measures of quantitative variation and 3D modelling of shells to show that the locus controlling band presence/absence mainly acts after band position is established. The work establishes a method that may be used for comparative studies of quantitative banding variation in snail shells, extraction of growth parameters, and morphometrics.

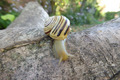

## 1 INTRODUCTION

The nature of shell growth in gastropods is useful because it preserves the ontogeny of shape, colour, and banding patterns, making them an ideal system for understanding how inherited variation develops and is established and maintained within a population (Johnson et al., 2019). This is particularly beneficial when considering animal coloration and patterning, both of which have been critical in understanding the key principles of evolution (Cuthill et al., 2017; Richards et al., 2013).

**FIGURE 1:**
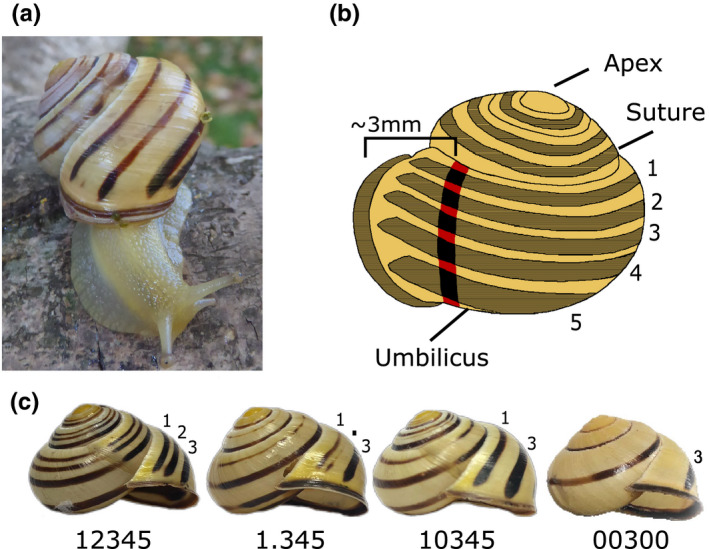
(a) *Cepaea nemoralis* from the Pyrenees, in this case a four‐banded form. (b) *Cepaea* shell showing shell characters and illustrating position for measurement of bands. c) Banding phenotypes considered in this study, from left: five bands (12345), missing second band (10345), partial missing second band (1.345), mid‐band (00300)

Historically, the foremost gastropod species in understanding colour polymorphism and band patterning has been the European land snail *Cepaea nemoralis* (Figure 1a), and its sister taxon *Cepaea hortensis* (Jones et al., 1977; Ożgo, 2011), partly due to their ease of collection. Also useful has been the ability to record morph frequencies, whether yellow, pink, or brown, with varying numbers of bands, from zero to five (Cain & Sheppard, 1950, 1952; Jones et al., 1977). A further reason is the apparent simplicity of the Mendelian inheritance of the shell colour and banding loci, many of which are inherited together in a ‘supergene’ (Cook, 1967; Jones et al., 1977). As a result, studies on the shell polymorphism of the snail *Cepaea* have played a crucial role in establishing the role of natural selection in maintaining morphological variation, with the genus becoming a pre‐eminent model for ecological genetics, alongside the peppered moth (Cook & Saccheri, 2013; Grant et al., 1996; Majerus et al., 2000; Walton & Stevens, 2018).

In the present day, one of the continuing benefits of working with *Cepaea* is an ability to compare the frequencies of shell morphs in historic collections against modern‐day samples, to infer the potential impact of natural selection and/or drift in changing shell morph frequencies (Arthur et al., 1993; Cameron, 1992; Cameron et al., 2013; Cook et al., 1999; Ożgo et al., 2017; Ożgo & Schilthuizen, 2012). Of particular use, the “Evolution Megalab” project digitized a large set of 20th‐century samples. These records, and others deposited in museums, are now being used with modern surveys to produce an increasing number of comparative papers (Cameron & Cook, 2012, 2013; Silvertown et al., 2011; Worthington et al., 2012). New studies on the genetics and genomics (Kerkvliet et al., 2017; Mann & Jackson, 2014; Richards et al., 2013; Saenko et al., 2021) mean that *Cepaea* snails are poised once again to become a powerful system. The findings from this single genus should lead the way in understanding the diverse variety of shell patterns that are found in the wider group of snails and molluscs to which they belong.

Unfortunately, a traditional focus on the qualitative scoring of the shell characters of *Cepaea* has resulted in a lack of knowledge regarding the mechanisms that control fine variation. For example, the ground colour of *Cepaea* has traditionally been grouped into one of three categories, yellow, pink, or brown. This was necessary for field‐based classifications, but recent spectroscopy and psychophysical modeling of avian visual systems has shown that the colour variation is continuously distributed, albeit around three clusters which roughly correspond to the qualitative colour groupings of yellow, pink, and brown (Davison et al., 2019). Although further studies are necessary, the observation of continuous variation in colour is intriguing because the traditional theory is that, provided observed variation results from frequency‐dependent selection, the underlying supergene that determines colour has evolved to prevent phenotypes from “dissolving” into continuous trait distributions. These findings raised questions about the nature of the selection that acts upon the polymorphisms.

With interest in quantitative variation in *Cepaea* colour (Davison et al., 2019), it seems appropriate to reconsider variation within and between banding patterns, which has received little attention since Rotarides (1926), who established that the proportion of shell covered by band is correlated with variation within habitat types. This and subsequent work using similar methods (Ożgo & Komorowska, 2009) have tended to focus on the proportion of the shell that is banded, and the potential effect on natural selection (Neiber & Hausdorf, 2015; Neiber et al., 2016). How the position and widths of bands might be established during shell growth has been neglected, yet could provide useful insight into how banding patterns vary within individual shells over time.

In banding notation (Cain, 1988), bands are numbered 1 to 5 from the top of the shell down, with modifications to recognize band fusions and interruptions (Figure 1b). A five‐banded snail with bands fused on the lower part of the shell is thus 123(45), and a mid‐banded shell is 00300. However, as with colour, the qualitative scoring of bands masks complexities. For example, a five‐banded individual may possess five wide bands which are close to fused with little ground colour visible between them, or it may possess five narrow bands, with considerable visible colour between the gaps. These individuals would be scored as having the same phenotype, yet the large differences between them may affect thermoregulation, visibility to predators, and resistance to crushing forces (Cook, 2008; Ożgo & Schilthuizen, 2012; Rosin et al., 2013; Staikou, 1999; Surmacki et al., 2013). Bands are integrated into the shell matrix, unlike colour which has no structural elements (Budd et al., 2014; Williams, 2017). In *Cepaea*, bands are present in all three layers of shell, and their presence in the central calcareous prismatic layer is likely responsible for the increased crushing resistance displayed by banded shells relative to their unbanded counterparts (Rosin et al., 2013).

How is band position determined? The main shell loci have been characterized but not yet identified. A locus *B* determines band presence/absence, locus *U* suppresses all bands except band 3 (to make a mid‐banded snail 00300), and another locus suppresses bands 1 and 2. Several other loci, including spread band *S* and punctate *I* (or ‘interrupted’) loci, modify the nature of the band phenotype. Individuals may also have unpigmented bands, a phenotype known as hyalozonate, where bands are present and visible, but lack the usual pigmentation, suggesting that while these processes may interact, the laying down of bands and the pigmentation of these bands occur independently of one another. There are also likely other loci, or environmental factors which act during growth, that exert a multifactorial effect on the phenotype, including modifiers of bandwidth, band fusion, band colour, suppression of individual bands, and the timing of band expression (e.g., bands only on last whorl). However, these loci are not useful in understanding how bands are placed, because they mainly specify presence/absence, or character, rather than position.

To begin to understand the genetic mechanisms underpinning pattern variation in *Cepaea*, a first step is to re‐evaluate the description of the banding phenotype by quantification of variation in banding patterns both between and within phenotypes, and throughout shell growth. Here, we combine empirical measures of quantitative variation within and between bands, and 3D shell models, to understand how bands are placed and interact with one another. By comparing fully banded individuals against shells lacking individual bands, we infer that the locus that controls band absence mainly acts after band position is established. We also show that the lower bands are not wider as an artifact of greater shell growth on the lower shell. They grow at the same rate as all other bands, but are wider from their first formation. Finally, we show that the same measures may be taken from a photograph, and a 3D model inferred. Validation of these methods for shell pattern quantification provides a baseline for future analysis of shell patterning and ornamentation in gastropods. As we move toward identifying the genes involved in setting the patterns, these findings may together be used to develop a model for band placement in snail shells, set in the general context of understanding shell growth parameters.

## 2 MATERIALS AND METHODS

### 2.1 Snails

Individuals of both species, *C. nemoralis* and *C. hortensis,* were collected by volunteers and on fieldtrips across Europe. Snails were euthanized by freezing at −80°C upon arrival at the University of Nottingham and subsequently thawed and bodies extracted from their shell.

Shell banding and colour phenotypes were first scored qualitatively, using the scheme described in Murray (1963), with some minor deviations where necessary (Davison et al., 2019). The main phenotypes of importance to this study were five‐banded, 12345, and mid‐banded, 00300 (Figure 1c). These were used to understand the impact of band absence on the position and width of band 3. In a Pyrenean population sample, shells lacking the second band, phenotype 10345, were relatively common. This population also included some shells in which band 2 was only present in the very last part of the shell, just before the lip. Here, we describe this feature as “.”, distinct from the mark used to represent punctate “:”, for example, 1.345. These shells were used to understand the impact of the absence of band 2, and also a partial suppression of band 2, upon the positions of the remaining bands.

### 2.2 Shell measurements

To measure the positions and widths of the bands on the *Cepaea* shells, a ~1 mm strip of electrical tape was wrapped around the last whorl of individual adult shells, from the suture to the umbilicus (Figure 1b). The tape was attached parallel to any growth lines and placed ~3 mm back from the shell lip, necessary because banding phenotype often differs close to the lip. Band start and end position were then recorded by marking the tape with a super‐fine permanent marker under a dissection microscope. Tape was removed from the shell, and the distances between marks were measured using Vernier calipers under a dissection microscope.

The individual measures of band position were converted into proportions, standardizing against the distance between the suture and the umbilicus, to enable comparison between shells of different sizes. The mid‐point of the band was used to define band position, with bandwidth considered separately. Individual measures were not used if bands were ill‐defined or fused. Shell height, width, and weight were also measured, to enable tests for associations with size, and shell shape (width/height).

### 2.3 Interactions between bands and bandgaps

We first checked whether other shell parameters influence band position and width. Statistical models were created, using height, weight, shape, and band position and width data, in R version 3.6.2. All full models included fixed effects of shell shape (obtained by dividing shell height by shell width), shell height (used as a proxy for shell size), and shell weight (as a proxy for shell thickness), as well as a random effect of population to remove this as a confounding variable. For model selection, a full set of models including every combination of fixed effects was generated. These models were ranked according to their Akaike information criterion (AIC). From a full model set, models with a value within two AICs of the best fitting model (value closest to zero) were considered to be equally supported, and so these were averaged. Full coefficients are quoted in the final averaged model, meaning that any terms not appearing in a given component model were assigned a coefficient of zero before averaging.

The null hypothesis was that if the deposition of pigment in each band is independent of others, then absence of individual bands in the adult shell will not impact upon the position and width of other bands. Mann–Whitney U tests were therefore performed to determine whether the position and width of band 3 varied in mid‐banded individuals (00300) compared with five‐banded individuals (12345) in *C. nemoralis*. Similarly, multivariate Kruskal–Wallis tests, followed by Dunn's pairwise tests with Benjamini–Hochberg adjustment, were carried out to determine whether partial or complete absence of band 2 impacted upon the position and width of the remaining bands.

Bands are established in juvenile snails, usually becoming progressively wider with each whorl of the shell. Bandwidth is necessarily constrained by the edges—the point of contact with the suture and toward the umbilicus—and likely also interactions with other bands, and the gaps between bands. Therefore, to understand how bands grow in width and interact with one another, the edges, and the gaps between bands, we tested all possible correlations between individual bandwidth and bandgap, focusing on the width of the gap immediately above or below each band. If bands increase in width together, a positive relationship will result between focal bandwidth and the widths other bands at the level of an individual snail. The corollary was an expectation for a positive relationship between individual bandgap width and other bandgap widths, and a negative relationship between bandwidth and bandgap width.

### 2.4 Comparison between species and colour

Differences in the position and width of each band between species were tested using five‐banded snails and generalized linear mixed‐effects models (GLMMs). Each band was modeled separately. Species was fitted as the sole fixed factor, with a random effect for population in each model. The fixed term of species was removed in each model, testing the effect of deletion by comparison of Akaike information criterion (AIC). The AIC of the GLMM including the fixed effect was compared with that of a generalized linear model without the random terms to provide an approximate test of the importance of population, as per Davison et al. (2019). As genes for colour and banding patterns of shells may be in linkage disequilibrium (Cook, 2005), GLMMs were repeated with colour as the sole fixed factor.

### 2.5 Shell growth and use of 3D models

Bands 3, 4, and 5 on a *Cepaea* shell are typically wider than bands 1 and 2. One explanation is that the wider bands are simply an artifact of greater relative growth on the lower part of the whorl. Therefore, two complementary methods were used to understand how bandwidth varies with growth of the final whorl.

Shell segments were removed with a small circular saw, in 90° increments until an entire whorl had been removed, at each of five points, measurements of bandwidth and position were taken as described above. In addition, shells were mounted on a flat surface with their apertures facing up, columella parallel to the surface. A photograph was also taken at each stage, ensuring that all bands were visible around the aperture. An updated version of the ShellShaper software (https://github.com/jslarsson/ShellShaper; Supplementary Methods) was used to build 3D models of shells, including the positions of bands, obtained by user‐defined landmarks from each of the 2D images as per Larsson et al. (2020). Models were based on three‐dimensional logarithmic helicospiral growth, although using only circular apertures and no shell thickness. Band position and width were defined for a predetermined number of bands on any given shell. Widths and positions were then extracted from the model and analyzed.

To determine whether growth rate was influenced by the position on the shell, GLMMs were performed on mid‐banded and five‐banded shells, with the response variable of growth rate, and a fixed effect of shell section, with a random factor of ID included to mitigate the potential differences between individuals. Least‐square means with Tukey adjustments for multiple comparisons were performed to allow direct comparison of shell areas to one another.

Comparative analysis was performed on the two methods using a Bland–Altman plot to analyze agreement between the two methods, using the average of paired measurements of five‐banded individuals for reference. Differences in measurements from each method at constant locations and stages of growth across shells were analyzed, and the measurement bias and 95% upper and lower confidence intervals found.

## 3 RESULTS

Band measurements were taken for 440 individuals, 271 *C. nemoralis* and 169 *C. hortensis*, across 40 populations, distributed throughout the UK and mainland Europe (Tables S1–S3). Shell shape, height, or weight did not impact upon the relative position or width of any of the five bands (Tables 1, 2). In each of the 10 final averaged models generated, one for each position and width of each band, no predictors were significant. Ten similar models were generated to test for associations of band position and width with shell ground colour. The sole fixed factor of colour was not a significant predictor of variance in any of the 10 models.

**TABLE 1:** Outcome of statistical tests for the impact of shell shape, height, or weight relative position of bands

Predictors	Band 1 position model				Band 2 position model				Band 3 position model				Band 4 position model				Band 5 position model			
	Coefficient	2.5% CI	97.5% CI	Weight	Coefficient	2.5% CI	97.5% CI	Weight	Coefficient	2.5% CI	97.5% CI	Weight	Coefficient	2.5% CI	97.5% CI	Weight	Coefficient	2.5% CI	97.5% CI	Weight
Intercept	**8.09**	3.87	12.32	—	7.88	−13.71	29.46	—	18.65	−6.42	43.71	—	**45.58**	37.21	53.96	—	1.41	−102.50	105.33	—
Shape	1.65	−3.54	6.84	0.45	12.21	−16.63	41.05	1	11.49	−21.86	44.85	1	2.37	−7.10	11.85	0.32	85.27	−49.04	219.58	1
Weight	0.13	−0.24	0.50	0.53	0.33	−0.81	1.47	0.58	1.00	−2.72	4.71	0.71	—	—	—	—	—	—	—	—
Height	−0.02	−0.14	0.09	0.24	0.08	−1.13	1.29	0.62	0.04	−1.31	1.38	0.58	−0.21	−0.54	0.12	0.77	2.54	−3.56	8.64	1
Height:Shape	—	—	—	—	−0.24	−1.86	1.38	0.14	−0.20	−1.97	1.57	0.11	—	—	—	—	−4.01	−11.88	3.87	0.36
Height:Weight	—	—	—	—	0.00	−0.05	0.04	0.1	−0.01	−0.10	0.07	0.13	—	—	—	—	—	—	—	—
Shape:Weight	—	—	—	—	—	—	—	—	−0.41	−4.55	3.72	0.1	—	—	—	—	—	—	—	—

Note

From a full model subset, models within two Akaike information criteria (AIC) of the best model were selected, and means of the coefficients were taken. All of the terms listed were included in all of the full models for each band position model. CI represents the confidence interval; weight represents the sum of weights from models in which the variable in question appears in the final averaged model. Coefficients in bold indicate those for which the 95% confidence interval does not include zero.

**TABLE 2:** Outcome of statistical tests for the impact of shell shape, height, or weight relative width of bands

Predictors	Band 1 width model				Band 2 width model				Band 3 width model				Band 4 width model				Band 5 width model			
	Coefficient	2.5% CI	97.5% CI	Weight	Coefficient	2.5% CI	97.5% CI	Weight	Coefficient	2.5% CI	97.5% CI	Weight	Coefficient	2.5% CI	97.5% CI	Weight	Coefficient	2.5% CI	97.5% CI	Weight
Intercept	**3.64**	1.363071	5.920491	—	**3.66**	2.23	5.10	—	**9.07**	3.36	14.78	—	**8.81**	5.24	12.38	—	10.34	−6.14	26.83	—
Shape	−0.69108	−3.48987	2.107718	0.32	—	—	—	—	—	—	—	—	−0.62	−4.50	3.26	0.18	−6.41	−33.19	20.37	0.21
Weight	0.02701	−0.11889	0.172911	0.24	0.11	−0.20	0.43	0.45	−2.12	−6.26	2.02	0.56	0.11	−0.32	0.55	0.34	−1.35	−7.59	4.88	0.39
Height	—	—	—	—	0.01	−0.05	0.08	0.2	−0.15	−0.46	0.16	0.56	−0.03	−0.17	0.11	0.47	0.23	−0.15	0.61	0.82
Height:Shape	—	—	—	—	—	—	—	—	—	—	—	—	—	—	—	—	—	—	—	—
Height:Weight	—	—	—	—	—	—	—	—	0.11	−0.10	0.33	0.56	—	—	—	—	−0.05	−0.27	0.16	0.18
Shape:Weight	—	—	—	—	—	—	—	—	—	—	—	—	—	—	—	—	2.98	−9.43	15.40	0.18

Note

From a full model subset, models within two Akaike information criteria (AIC) of the best model were selected, and means of the coefficients were taken. All of the terms listed were included in all of the full models for each band position model, but several model averages include a reduced model with no fixed factors. CI represents the confidence interval; weight represents the sum of weights from models in which the variable in question appears in the final averaged model. Coefficients in bold indicate those for which the 95% confidence interval does not include zero.

### 3.1 Effect of missing bands

**FIGURE 2:**
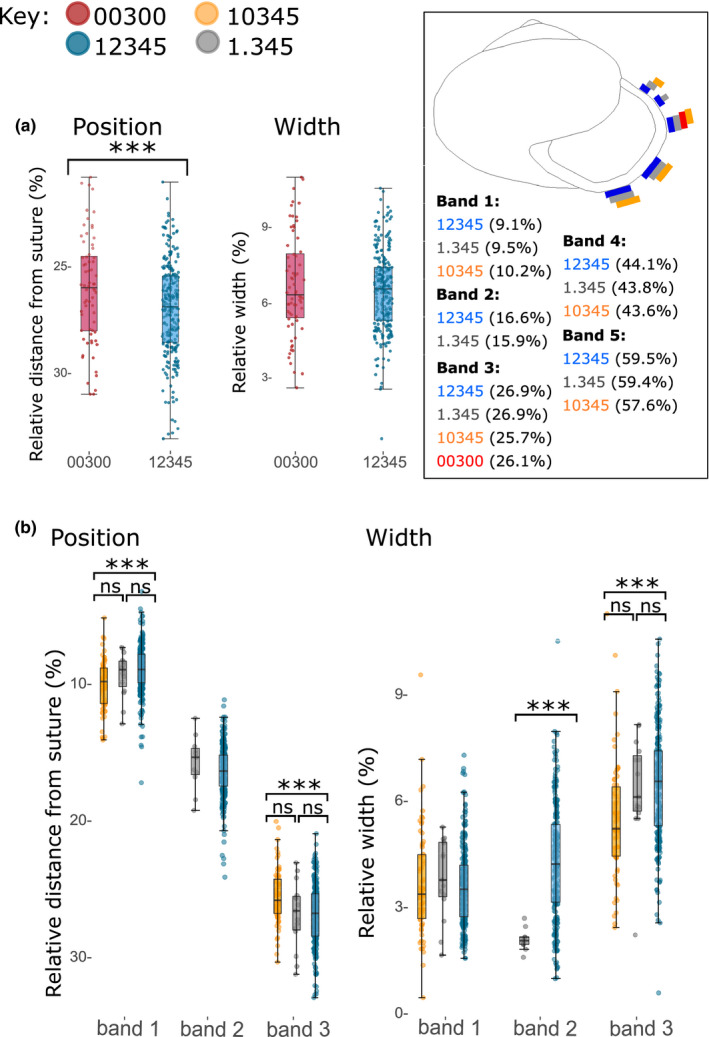
Band positions and widths in different phenotypes. (a) Band 3 in mid‐banded (00300) individuals is shifted ~0.9% upward compared with the same band in five‐banded (12345) snails. The width of band 3 does not differ between the same phenotypes. (b) In shells in which band 2 is missing (10345), bands 1 and 3 are ~2.4% closer together. There are also some differences in bandwidth, especially band 3. *p* < 0.05,*; *p* < 0.0001,***. Inset: summary of band positions in different phenotypes

Mann–Whitney *U* tests demonstrated that, in *C. nemoralis*, when other bands are absent, the mid‐band was shifted toward the top of the shell, albeit only ~0.9% closer (*W* = 6,867.5, *p* = 0.0107; Figure 2a). In comparison, the mean difference between first and second measures of the same band was 0.17%, ranging between 0.004% and 0.7%. The absence of other bands did not impact upon the variability in position of the band of a mid‐banded individual; Kolmogorov–Smirnov tests demonstrated that distributions were equal when shifted to center around a single mean, suggesting that variance in band position remained constant in both phenotypes (*D* = 0.08, *p* = 0.9). The width of the bands also did not change in the absence of other bands (*W* = 8,831, *p* = 0.7; Figure 2a). Gaussian finite mixture modeling of the distribution of widths indicated that the width of band 3 in five‐banded individuals is not multimodal. Both the best model (X, univariate normal, BIC −295.4; *p* = 0.04 compared to second best model) and the next best models resolved a single cluster. As with band position, the distribution of bandwidths in mid‐banded snails did not differ from the distribution of individuals with five bands.

Similarly, Kruskal–Wallis tests indicated that when band 2 was missing or partially suppressed (Figure 2b), both bands 1 and 3 were in different positions across the three phenotypes (*H* = 18.05, *df* = 2, *p* = 0.0001; *H* = 17.1, *df* = 2, *p* = 0.0002). Specifically, bands 1 and 3 were ~2.4% closer to each other when band 2 was absent (Figure 2b). Pairwise Dunn's tests with Benjamini–Hochberg adjustments indicate that this difference was only present between the 12345 and 10345 phenotypes for both bands one and three (*Z* = −4.1, *p* = 0.000007; *Z* = −4.2, *p* = 0.0001), with the partially suppressed phenotype intermediate and nonsignificantly different from the bands 1 and 3 in 10345 (10345; *Z* = −1.4, *p* = 0.2; *Z* = 1.9, *p* = 0.06), and 12345 (*Z* = 0.6, *p* = 0.5; *Z* = 0.09, *p* = 0.9). Band 4 was in a consistent position, but band 5 was shifted upward, by ~1.8%, in the absence of band 2 (*Z* = −3.0, *p* = 0.0009); band 5 was in the same position in shells of phenotype 12345 and 1.345.

Kruskal–Wallis tests indicated that band 1 did not differ in width across the three phenotypes (*H* = 1.2, *df* = 2, *p* = 0.6), whereas band 3 width did differ (*H* = 23.1, *df* = 2, *p* = 0.00001). Pairwise Dunn's tests with Benjamini–Hochberg adjustments indicated that there was no difference between any of the phenotypes in band 1 (*Z* = 1.02, *p* = 0.3; *Z* = 1.1, *p* = 0.3; *Z* = −0.1, *p* = 0.9). The width of band 3 differed between 12345 and 10345 phenotypes (*Z* = −4.8, *p* = 0.000005), with band 3 narrower when band 2 was absent. No difference in the width of band 3 was observed between the other phenotypes (*Z* = 2.3, *p* = 0.06; *Z* = 0.05, *p* = 0.96). The width of band 2 varied significantly between the partially suppressed phenotype and 12345 individuals (*H* = 20.6, *p* = 0.000006).

### 3.2 Interactions between bands and bandgaps

**FIGURE 3:**
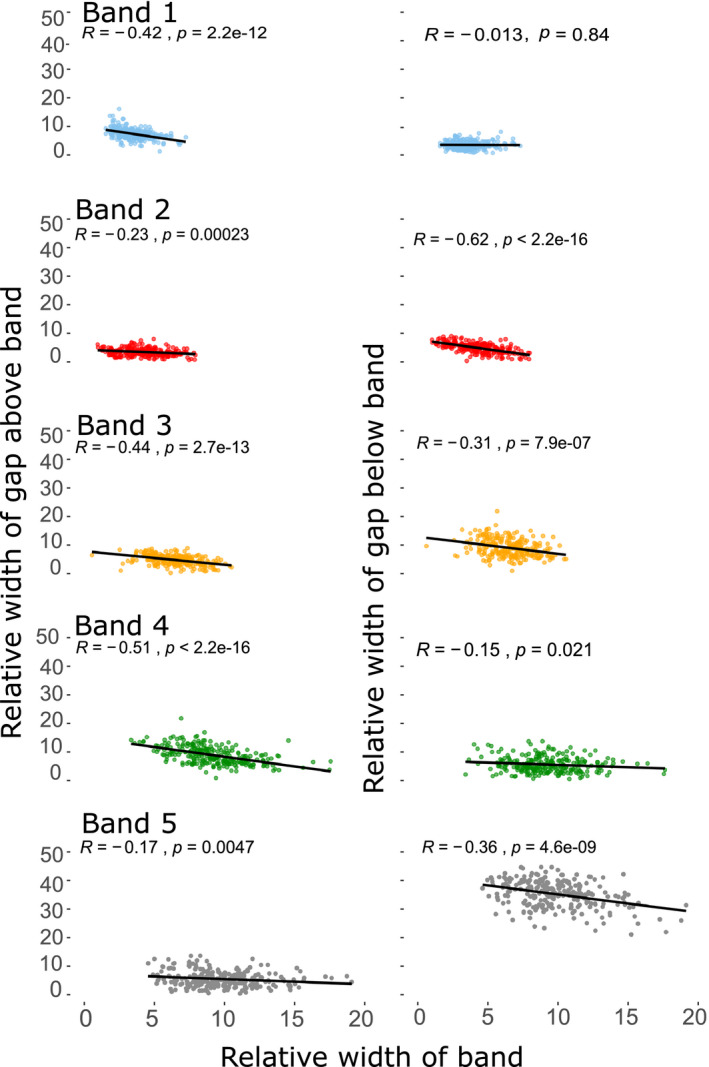
The relationship between the width of a band and the widths of the gap above and the gap (left‐hand side), and below (right‐hand side) in five‐banded *Cepaea nemoralis*. Most of the correlations are significantly negative, as expected if bands expand in width by occupying the gaps in‐between

When individual bands were larger, the corresponding gaps above the band tended to be smaller (Figure 3), with band 4 showing the strongest relationship (*R* = −0.5, *p* < 2.2e−16), and band 5 the weakest (*R* = −0.2, *p* = 0.005). The same relationship was found between the individual bands and the gap width below (Figure 3); except that band 2 showed the strongest relationship (*R* = −0.6, *p* < 2.2e−16) and band 1 did not show any correlation with the band below (*R* = −0.01, *p* = 0.8).

**FIGURE 4:**
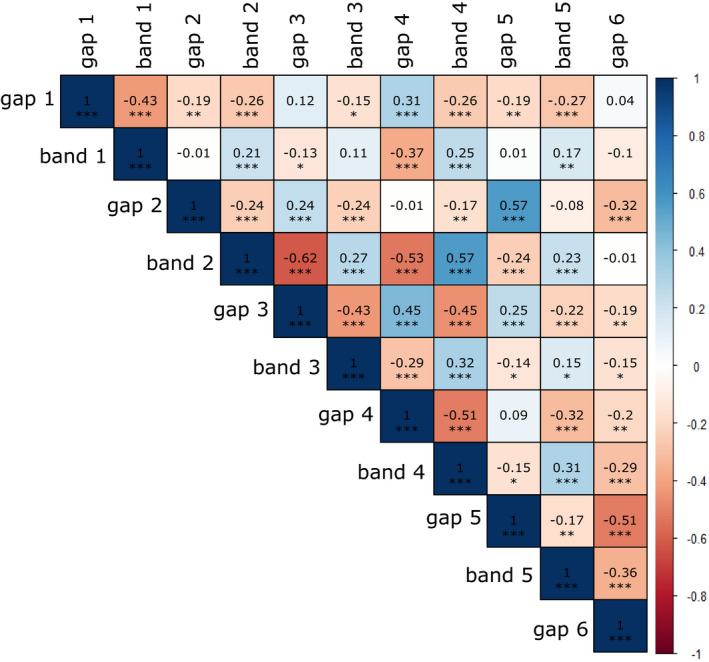
Matrix showing correlation between the width of all bands and the width of all gaps, where gap 1 is the gap preceding band 1, next to the suture. Positive relationships are shown in shades of blue and negative relationships in shades of red. *p* < 0.05, **; *p* < 0.01; ***p < 0.001

In testing all comparisons between bandwidths and bandgap widths, most relationships were in the expected direction, except for some of the gap–gap comparisons (Figure 4); there were unexpected negative correlations between gaps 1/2 (*R* = −0.2, *p* = 0.004), 1/5 (*R* = −0.2, *p* = 0.003), 2/6 (*R* = −0.3, *p* = 0.000003), 3/6 (*R* = −0.2, *p* = 0.003), and 5/6 (*R* = −0.5, *p* < 2.2e−16).

### 3.3 Comparison between species

**FIGURE 5:**
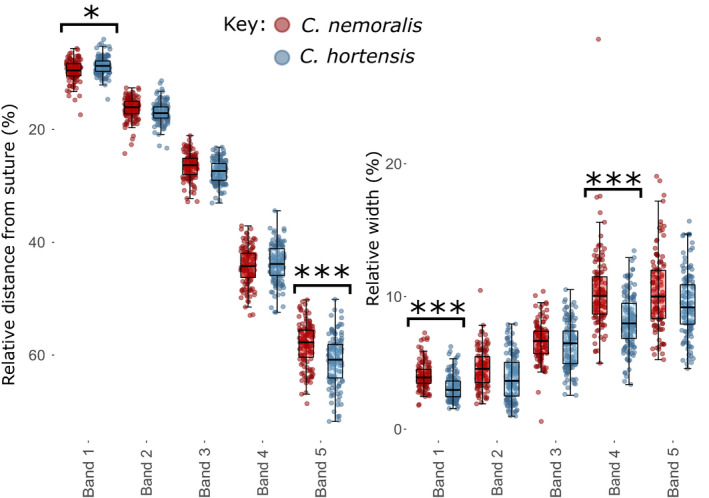
FIGURE 5 Between species comparison of the position (left) and width (right) of each of the five bands in five‐banded individuals

The bands had broadly similar positions and widths in the two species, with some minor, significant differences in magnitude (Figure 5). In *C. nemoralis,* band 1 was ~1% toward the base of the shell, whereas band 5 was ~3% closer to the top (*X* ^2^ = 4.4, *df* = 1, *p* = 0.04; *X* ^2^ = 12.6, *df* = 1, *p* = 0.0004). *C. nemoralis* individuals also had slightly narrower bands in positions 1 and 4 compared with *C. hortensis* (*X* ^2^ = 18.05, *df* = 1, *p* = 0.00002; *X* ^2^ = 21.8, *df* = 1, *p* = 0.00003).

### 3.4 Shell growth and use of 3D models

**FIGURE 6:**
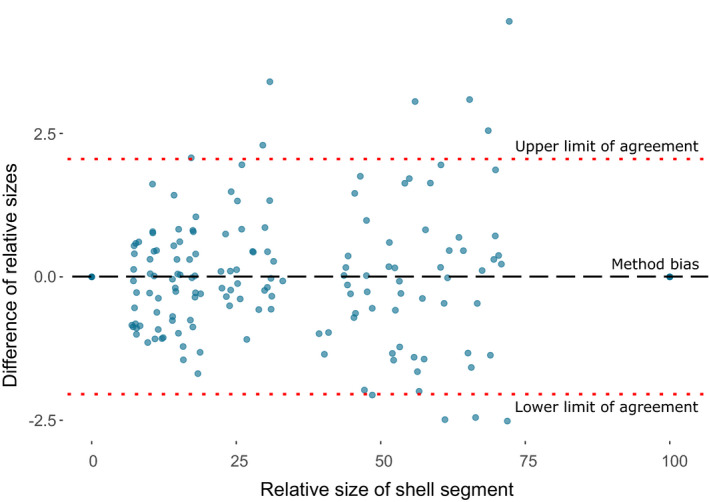
Bland–Altman plot of relative widths of shell sections of five‐banded individuals. *X*‐axis represents the average measure of width of shell segment taken by the two methods, and the *y*‐axis represents the difference of measurements from this average. The line of bias (black dashed lined) and the 95% limits of agreement (red dotted lines) are shown

Bland–Altman plots of paired shell measurements (Figure 6) showed that neither the tape or computer‐based method resulted in measurements which were consistently larger or smaller than the other; thus, the differences in the plots show data points scattered evenly above and below zero. There was no consistent bias between the two methods (Bias = 0.005), and 95% of the data fell between the upper and lower limits of agreement of −2.04 and 2.05. This confirmed that while there is variation, the model is able to reproduce the 3D shape from a 2D photo, and also, that ShellShaper is able to extract band‐measurement data from a 2D image, while retaining information revealed by manual measurements.

**FIGURE 7:**
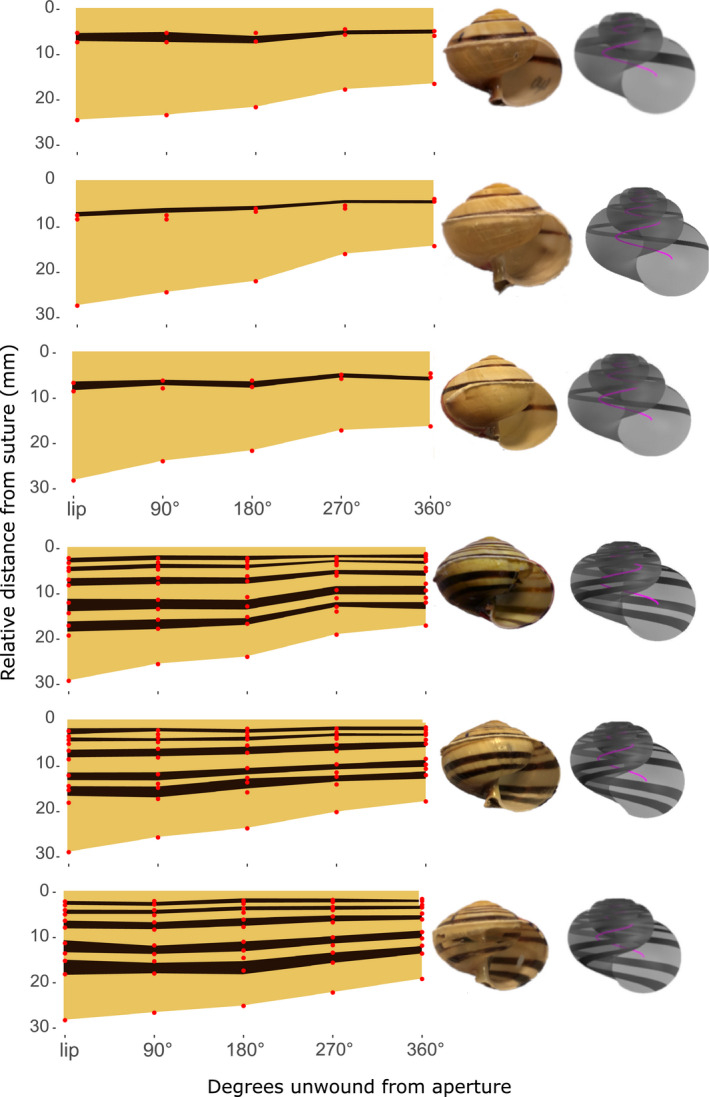
Projection of band position and width over last whorl of shell, using mid‐banded (top three), and five‐banded (bottom three) individuals. Manual (red points) and ShellShaper (dark shading) inferred measures show the same patterns. Also shown is a photo of each shell, and a 3D model generated by ShellShaper

Models fitted with fixed effect of shell region, and random effects for distance along the last whorl, and individual, demonstrated that regions of shell in both mid‐banded and five‐banded shells grow at different rates (Figure 6; *X* ^2^ = 119.7, *df* = 10, *p* < 0.0001; *Χ* ^2^ = 84.9, *df* = 2, *p* < 0.0001). Pairwise comparisons show that this difference is exclusively between all shell regions and the region between the last band and the umbilicus. The bottommost area grows at a faster rate than other areas of the shell, which all increase in size at an equal rate throughout growth (Tables 3, 4). The relative proportions of the shell covered by each region changed along the whorl, as the lowermost region of the shell expanded more rapidly than the others. All other shell regions remained at equal proportions relative to one another throughout growth (Figure 7). Models were repeated with distance along the last whorl as the sole fixed factor, with random effects for shell region and individual. These demonstrated that there is no difference in growth rates in areas of the shell across the length of the last whorl in five‐banded or mid‐banded snails (mid‐banded: chi‐squared = 0, *df* = 10, *p* = 1; five‐banded: chi‐squared = 0, *df* = 10, *p* = 1). Expansion per quarter whorl in every shell section remains constant throughout the growth of the entire last whorl.

**TABLE 3:** Pairwise comparisons of proportionate differences in growth rates between areas of shell in mid‐banded individuals

Comparison		Estimate	*SE*	*df*	*t*‐ratio	*p*‐value
Gap 1	Band 3	1.9537	1.0503	36.893	1.8601	0.1647
Gap 1	Gap 2	−1.3259	1.0503	36.893	−1.262	0.4251
Band 3	Gap 2	−3.2796	1.0503	36.893	−3.122	0.0094

Note

Data generated by construction of 3D ShellShaper models.

**TABLE 4:** Pairwise comparisons of proportionate differences in growth rates between regions of shell in five‐banded individuals

Comparison		Estimate	*SE*	*df*	*t*‐ratio	*p*‐value
Gap 1	Gap 6	−3.43	0.79	141.62	−4.32	0.0014
Band 1	Gap 6	−3.18	0.79	141.62	−4.01	0.0046
Gap 2	Gap 6	−3.32	0.79	141.62	−4.18	0.0025
Band 2	Gap 6	−3.10	0.79	141.62	−3.90	0.0067
Gap 3	Gap 6	−3.67	0.79	141.62	−4.62	0.0004
Band 3	Gap 6	−3.15	0.79	141.62	−3.97	0.0053
Gap 4	Gap 6	−4.05	0.79	141.62	−5.10	0.0001
Band 4	Gap 6	−3.38	0.79	141.62	−4.26	0.0018
Gap 5	Gap 6	−3.65	0.79	141.62	−4.60	0.0005
Band 5	Gap 6	−3.06	0.79	141.62	−3.85	0.0080

Note

Data generated by construction of 3D ShellShaper models. Only significant comparisons included.

### 3.5 Allometric shell growth

In order to produce the convex spires seen in globose species such as *Cepaea,* allometric growth is necessary. The type of allometry needed for this requires an increase in height of a complete whorl being greater than the increase in width of the same whorl. To confirm the required type of allometry was present in growing shells, a basic allometry test was used to determine whether that the growth in width was smaller than the growth of the height in the shells measured with ShellShaper. Wilcoxon signed‐rank tests indicate that the increase in whorl height is greater than the increase in whorl width (*V* = 465, *p* = 0.00000009), confirming the allometric growth parameters necessary to produce a convex spire.

## 4 DISCUSSION

In the past, the banding phenotype of *Cepaea* snails has typically been scored as a qualitative character, even though shells with the same number of bands may have a quite different outward appearance. Here, we developed a method to describe quantitative variation in the banding patterns of both species and then use these findings to test the interactions within and between bands and other shell characters. Broadly, we found that the precise position of bands depends upon the presence or absence of other bands, although the effect size is small. These findings give a first hint of the pathway that defines the positions and pigmentation of bands in the shell. By comparing the method with inferences from a 3D model, we show that the same quantitative measures may be applied to a 2D photo of a shell. Overall, the findings provide a starting point for exploration of how bands are placed in *Cepaea*, and the origins of fine variation in banding pattern.

### 4.1 Pigmentation of individual bands is independent

If the deposition of pigment in each band is independent of other bands, then one argument is that absence of individual bands in the adult shell should not impact upon the position or width of other bands. However, if there are fewer bands, then the absolute position of the remaining bands becomes of less importance, provided they do not overlap. Band position might then vary slightly, or the width might show greater variation in the absence of other bands. For example, a predator will tend to see a single mid‐band, irrespective of the precise position on the shell. In comparison, in a five‐banded snail, the mid‐band must be distinct from the other bands (unless there is a genetically coded band fusion), which reduces the range of possible positions.

In comparisons between the position of the third band in mid‐banded and five‐banded shells, we found that the band positions were broadly the same. This was also true of comparisons between the positions of the first and third bands in individuals where the second band was present or absent. Bands occupied more or less the same shell space as the corresponding band in a fully banded snail and did not cross over into the space which the other bands normally occupy. Yet, there were some small but significant differences in position. For example, the second and third bands were typically found at 16.6% and 27.0% of the distance from the suture (Figure 2 inset); in mid‐banded snails, the third band was slightly closer, 26.1%, to the suture. Similarly, the first and third bands were typically found 9.1% and 27.0% (as before) from the suture. When band 2 was missing, bands 1 and 3 were closer together, 10.2% and 25.6% from the suture. Shells with a band 2 that was only present on the last part of the shell were intermediate for the position of bands 1 and 3. In comparison, we did not find any difference in the widths of any of the bands when other bands were absent, nor any evidence that the differences are influenced by shape or ground colour of the shell. These results therefore show that while the approximate position of the bands is the same, there is a limited degree of lability in their placement that is contingent upon the presence or absence of other bands.

There are two main explanations for these findings. The first is that the position of all five bands is established and maintained early in shell development, even in the absence of individual bands. The spatial signal for the five bands is likely present in a molecular sense, but the pigmentation is lacking for individual bands. This would imply that the locus for band absence acts late in the pathway that establishes bands. An alternative explanation is that individual band positions are established independently of each other, such that if one band is not present, then this does not impact upon the position of others. In this case, individual band position would have to be defined relative to a fixed character, such as the suture. In our opinion, this second explanation is less credible because we found evidence that the bands do interact, at least to a small degree. Bands differed slightly in position when other bands are absent, including evidence that even latestage band expression can interfere with the position (Figure 2). More generally, if bands do not interact, it is difficult to understand why instances of mis‐positioning of bands were not more common. An analysis of hyalozonate patterns compared against those displaying fully pigmented bands could shed light on the relationship between pattern establishment and pigmentation.

To further explore how bands are placed and interact with one another and shell edges, we investigated correlations between the bandwidths and the gaps between bands. This was also partly motivated by wanting to understand the reason that bands 3, 4, and 5 are consistently wider than bands 1 and 2. The temptation might be to put the differences down to natural selection, but the default explanation must be nonadaptive. For example, perhaps the topmost bands are narrow because they are constrained by the suture edge. Alternatively, the bottommost bands might be wider because their expansion is correlated with growth of the expanding whorl on the lower part of the shell, and band widening is simply an artifact of the deposition of new shell material.

Broadly speaking, the results showed that bands expand in width at the same rate. Where bands were wider in adult shells, the corresponding gap above and below each band was narrower (Figure 3). There were some unexpected slight negative correlations between the first gap (next to the suture) and the first band with other bandgaps, as well as negative correlations between the last gap (next to the umbilicus) and some other bandgaps. As the negative correlations mainly involved edges, then perhaps the bandgaps at the edges indirectly exert some effect to maintain a narrow gap between the band and the edge?

Moreover, the projections that were taken from manual measurements (Figure 3) and those inferred from 3D models (Figure 7) confirmed that all of the regions of the shell expand at the same rate, with the exception of the lowermost part of the shell, the final bandgap before the umbilicus (Figure 7, Tables 3, 4). The widths of the bands are significantly correlated for bands 3, 4, and 5 (*R* = −0.2, −0.3, −0.4, all *p* < 0.001; Figure 4), such that as an individual band gets wider, then the last bandgap gets proportionately narrower. However, there is no such relationship for bands 1 and 2 (*R* = −0.01, −0.1, neither significant).

Although all bands and the gaps between them become progressively wider, the last gap (i.e., the gap between the end of the final band and the umbilicus) expands at a faster rate than the rest of the whorl. This implies that the lower bands are not simply wider as an artifact of shell material deposition during growth, but rather that the lower bands start wider, and so remain wider throughout growth. The consistency of growth rates across all bands, and therefore the gaps between them, suggests that the widths of all bands are under similar mechanisms of control/constraint, irrelevant of their position on the shell. The increased growth rate of the lowermost part of the shell is perhaps simply due to the relative downward movement of the aperture in the allometric growth necessary to produce shells with a globose spire, such as *Cepaea*. It is perhaps also likely that the final bandgap becomes larger with shell growth due to a change in the generating curve in the final growth stages of the shell, where the angle of the aperture of an adult shell is further from vertical than in juveniles.

### 4.2 3D models to infer band position and shell shape parameters

The initial method used to measure bands used electrical tape and a dissecting microscope. This means that it was straightforward, but also laborious, difficult to scale, and limited in the data that were collected. These issues were resolved using ShellShaper software. By taking a 2D photo of a shell with the aperture facing upward, ShellShaper was used to take the same band position measures and also to make 3D reconstructions of the shell (Figure 7). While the measurements were varied (95% limits of agreement of ~2% in either direction), there was very limited bias between the two methods, suggesting that neither method consistently under or overestimated the size of a shell segment. While larger sections of the shell (i.e., those toward the umbilicus) appear to produce more variable results when comparing the two methods (Figure 6), this may simply be due to the very different nature of the two methods, and inevitable slight differences in exact measurement position or angle of an area which grows more rapidly than the rest of the shell. The overarching patterns remain constant between the two methods, despite small discrepancies in exact measurements of individual segments.

Using ShellShaper has the advantage that the method may be applied to species with smaller shells, and those with more bands than *Cepaea*. The method also generates a shell model that can be used for further analyses, including the extraction of growth parameters that will allow for investigations of the similarities and differences within and between many different species of gastropods. Using ShellShaper for such comparisons would allow higher throughput data collection, allowing the collection of much larger datasets in both comparative and species‐specific studies. While ShellShaper allows comparison of bands in a context similar to traditional geometric morphometrics, the version used here works on the assumption of circular apertures, limiting its use in understanding how band patterns might change in relation to the shape of the aperture or other shell characters. Continuing development and increasing sophistication of 3D models produced by ShellShaper, means that such analysis with the use of varying aperture shapes is a possibility in the future. Complementary methods devised by others (e.g., Liew & Schilthuizen, 2016) may also be used for the same function, and be more suitable, especially when there is great variability in shell form. Other methods require complex, time‐consuming, and expensive techniques, such as CT scanning. ShellShaper has the advantage that a 3D structure can be generated from a single 2D photograph of the shell, which allows for relatively high throughput. While other methods include options such as producing models with noncircular apertures and external shell ornamentation, the ease of inclusion of analysis of banding position and size in ShellShaper provides added advantages not present in other methods.

### 4.3 Interspecies variation

The banding patterns were broadly similar in the two species of *Cepaea*, albeit with some small differences. For example, bands 1 and 4 were narrower in *C. nemoralis*, and band 1 was closer to band 2, and band 5 closer to band 4. These results indicate that control of band deposition mechanisms is only subtly diverged in the two species. Such slight differences in phenotype are unlikely to be detectable to avian predators, although this requires experimental confirmation (Davison et al., 2019; Delhey et al., 2015). Understanding the variation, or lack thereof, present in these banding patterns does, however, provide a starting point in establishing the underpinning genetic mechanism, including in relation to other species.

### 4.4 Reaction diffusion mechanism

The underlying mechanisms behind both the formation, and the control of the position and widths of the bands, in *Cepaea* remain unexplored. Although the reaction–diffusion model has been hypothesized to be of importance in pattern formation in other organisms (Gravan & Lahoz‐Beltra, 2004; Kondo, 2002), the interpretation of the models underlying shell pigmentation is limited to mathematical modeling of hypothetical signaling events (Budd et al., 2014). The models assume that pigmentation is caused by localized excitation and inhibition operating along a line of cells at the mantle edge during biomineralization. It is not currently known whether the cells involved in pigment secretion are organized in this manner. The precise identity of the molecules involved in molluscan pigmentation also remains relatively uncertain (Budd et al., 2014). To date, there is no definitive evidence that the banding in *Cepaea* is under the control of the reaction–diffusion model.

In several land snail species, including *Cepaea*, the same pigmentation patterns can be observed on both the shell and the mantle (Emberton, 1963). The presence of bands on the mantle suggests that the system controlling pigmentation may not be controlled by the simple “line of cells” as first assumed. It should be noted also that physical cues in marine gastropod shells possessing varices (thickened protrusions of shell) do not appear to be the main mechanism used to position new shell structures. Instead, it has been suggested that positional information of these structures is created by a Turing‐like system, but with previous shell structures providing some fine‐tuning feedback (Webster & Palmer, 2019).

While it may be hypothesized that Turing's reaction–diffusion model plays a role in the formation of shell patterns in molluscs, identification of the genes is a first step before testing whether the interacting substances are necessary in defining the patterns. We envisage two converging routes by which this may be made possible, either taking a gene mapping and pattern‐led approach (Cossins et al., 2006; Harper et al., 2011; Peichel & Marques, 2017), or else by comparing spatial gene expression (Adamson et al., 2017; Landgrebe et al., 2002; Ståhl et al., 2016).

It will certainly be interesting to investigate gene expression in relation to the wide diversity of shell phenotypes. For example, it is conceivable that unbanded *Cepaea* still contain the spatial molecular markers that correspond to bands, but that they are not pigmented—if that is the case then any subtractive method (comparing gene expression in banded vs. unbanded snails) will not work. To date, proteomic and transcriptomic studies have begun to identify both novel and co‐opted ancient genes involved in biomineralization and shell deposition (Clark et al., 2010; Jackson et al., 2010; Joubert et al., 2010; Mann & Jackson, 2014; Marie et al., 2013), which may ultimately assist in elucidating the formation and maintenance of variation within and between banding phenotypes in *Cepaea*.

Overall, by establishing a method for quantitatively measuring variation in an established banding pattern, and beginning to characterize pigments present in the bands, this work provides a baseline for further studies on the *Cepaea* banding polymorphism. This is true both from the perspective of understanding the presence and maintenance of variation in these banding patterns, and ultimately, the underpinning genetics involved. A next step must be to identify the component parts and evolutionary origins of the supergene in *C. nemoralis* and *C. hortensis*. A recent genome assembly is a first step toward achieving this aim (Saenko et al., 2021).

## CONFLICT OF INTEREST

The authors have no conflict of interest to declare.

## AUTHOR CONTRIBUTIONS

**Hannah J. Jackson:** Conceptualization (equal); Data curation (equal); Formal analysis (equal); Investigation (equal); Methodology (equal); Resources (equal); Validation (equal); Visualization (equal); Writing‐review & editing (equal). **Jenny Larsson:** Formal analysis (equal); Investigation (equal); Methodology (equal); Resources (equal); Software (lead); Validation (equal); Visualization (equal); Writing‐review & editing (equal). **Angus Davison:** Conceptualization (equal); Formal analysis (equal); Funding acquisition (equal); Investigation (equal); Methodology (equal); Project administration (equal); Resources (equal); Supervision (equal); Validation (equal); Writing‐original draft (equal); Writing‐review & editing (equal).

## Supporting information

Table S1‐S3

Supplementary Material

## Data Availability

All data for this manuscript are supplied in the Supplementary Information.
