# Supplementary material for: Quantitative measures and 3D shell models reveal interactions between bands and their position on growing snail shells
Source: Ecol Evol. 2021 May 2;11(11):6634–48. doi: 10.1002/ece3.7517 (PMC8207382; doi:10.1002/ece3.7517)
Supplement: Supplementary file 2 — Supplementary Material [file ECE3-11-6634-s001.pdf]

ShellShaper user guide
Banded version for circular apertures

Jenny Larsson
jslarsson1@sheffield.ac.uk

2020-11-24

ShellShaper is a program for extracting information about shape and banding position in snail shells from standardised photographs. Shells in the images need to be in the correct orientation and this banded version currently only works when apertures are circular.

1 Preparation

- You need to have access to MATLAB (`www.mathworks.com`, version R2018b or later). Including the curve fitting toolbox, and image processing toolbox. You do not need to be proficient in MATLAB to use ShellShaper.
- The ShellShaper program files can be found here: `https://github.com/jslarsson/ShellShaper`
- Preferably save the downloaded ShellShaper folder as a subfolder of your MATLAB folder.
- Prepare one folder to include the shell photos you want to analyse, and create one folder for the output.

2 Setup

1. Start MATLAB
2. Change directory to the folder where you saved the ShellShaper scripts and open the following file:

   `ShellShaperBands.m`

3. Change all paths of folders and files to ones that are correct for your file setup. All folders need to have been created before running the program. Make sure the paths are correct and does not overwrite already existing files unless that is your intention, MATLAB will overwrite files without asking for permission.
4. You can choose to only analyse a subset of the images by changing the 'startNumber' and 'lastNumber', they will be numbered alphabetically as found in the folder. This is useful for when there are more images than you can reasonably process in one go.
5. There are two settings for visuals. You can choose if you want the internal spiral to be visible, and if the colour scheme should represent orange *Cepaea* shells, or be greyscale, see figure 1f.

3 Running the program

Run the program by either pressing F5 or the big green arrow button in the tool strip. Make sure to do the above setup first, and that the current directory is set to the folder containing the ShellShaper scripts.

1. First the program will ask you to input the maximum number of bands found in the group of shells in your analysis.
2. A new window opens containing an image from the selected image folder, and a dialogue box pops up asking you to select the numbers of all bands that are present in this particular specimen, starting from the top by the suture and numbered clockwise.
3. Click on the image to position the first point at the apex, you can drag points around after initial placement. Press Enter when done.
4. Position the right and left extreme points of the latest whorls similar to figure 1b, pressing Enter will let you position the next one.
5. Position and resize the circle, see figure 1b, press Enter.
6. Position the aperture end points, one at the suture and one at the columnella, press Enter.
7. Place the start and end points for each of the present bands in clockwise order starting from the suture, see figure 1c. Press Enter after each is positioned.
8. To get the scale correct, find a known distance by moving the endpoints of the line L, press Enter, and input the value in the dialogue box, see figure 1d.
9. There will be a pink shell model on top of the original image, see figure 1e, as well as a second window opening with a grey or orange shell model by itself, see figure 1f. A dialogue window will open on top of the image asking if the model is good enough. You have 2 options:

   Yes This saves images of model and model on original image and the relevant parameters, and lets you go to next one.

   No This will let you reposition all the objects to find a better approximation. This will also let you rotate the model to inspect it further and save other orientations as images. Press Enter to see the updated models, if it looks good enough, then press yes in the pop-up instead, otherwise press no again and reposition the points again.
10. The name and number of the image just analysed is shown in the command prompt, and the result is saved to the .txt-file. The next image in the folder will open automatically, repeat step 2-9 for each image.
11. When 'lastNumber' has been reached, the program will close the image windows and output 'Done!' in the command prompt.

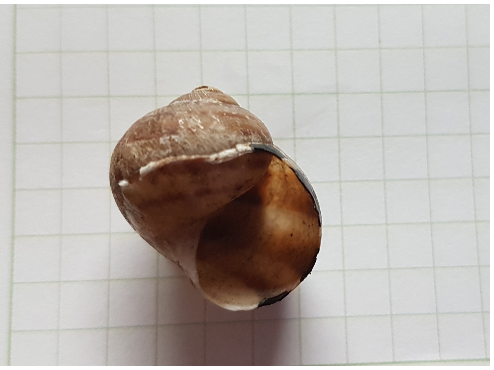

(a) Original image.

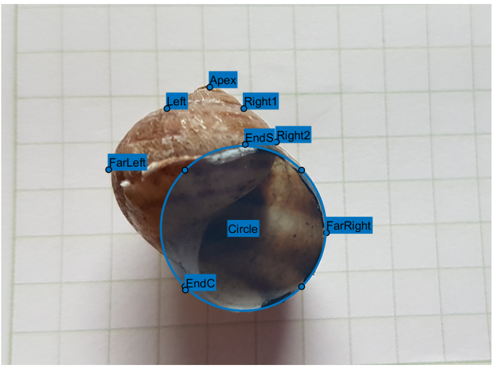

(b) Positioning the shape points and circle.

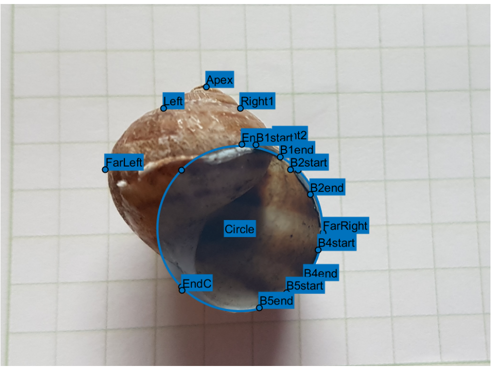

(c) Positioning the band points.

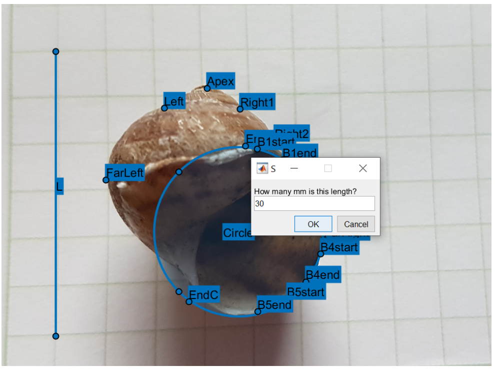

(d) Add size input.

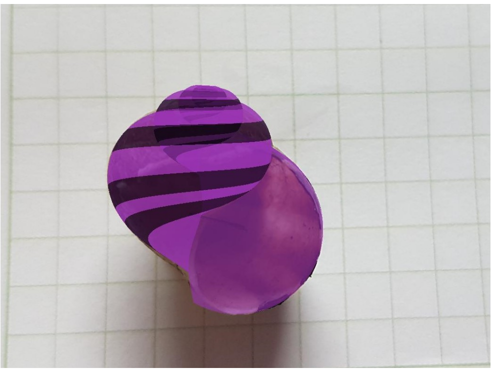

(e) Model with bands visualised on original image.

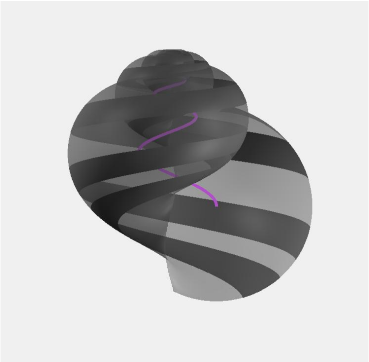

(f) Semi-transparent model with internal spiral in pink.

Figure 1: Example of procedure. Input image (a), interactive positioning of objects (b)-(d), output images (e)-(f).
